# Excerpts from the Case-Book of T. S. Powell, M. D., Sparta, Georgia

**Published:** 1857-03

**Authors:** 


### ARTICLE III.

*Excerpts from the Case-Book of* T. S. POWELL, M. D., Sparta, Georgia.

CASE 1.—*Protracted Constipation.*—On the 10th June, 185?, I was desired by Mr. P. to see his wife as soon as possible—I saw Mrs. P. in about 15 minutes; found her sitting on the bed-chamber, in a state of suffering difficult to describe—she was screaming loud enough to be heard from any part of the house; pulse scarcely to be felt, and body covered with a clammy sweat.

Under these circumstances, I dissolved gr. iss. ext. belladonna, in ℥i. warm water, and with a P P syringe, injected one-half; an hour afterwards, the second half was injected, which was soon followed by copious evacuations from the bowels, pulse rose, pain ceased, and in a few hours Mrs. P. felt quite restored. Ordered gr. ii. blue mass to be taken every night for a week.

CASE 2.—On the evening of the 22d of Sept., 1855, I was called to Mr. J. B., an elderly gentleman, 65 years of age. He complained of excruciating pain in the abdomen, with some nausea, which I attributed to hardened fecal matter in the bowels, as he informed me that he had not had an evacuation in 10 or 12 days. He had taken several doses of castor oil in the last two days, which was rejected. The pains came on periodically—pulse 95, full and sharp—prescribed calomel, gr. x, dov. powd., gr. v; hot fomentation to the abdomen. I called again in a few hours; the medicine had been retained,but had no effect—complains of great pain in the bowels, ever and anon. Prescribed, gr. ii ext. belladonna, sol. in ℥ii warm water, one-half to be injected immediately. Called again in two hours, found Mr. B. quite easy; has had several large evacuations of dark, hardened fecal matter. Ordered gr. ii blue mass every night for a week, and a seidlitz powder for three mornings.

Case 3.—*Tic Doloureux.*—In the spring of 1853, I was called to see Miss C., a beautiful and robust young lady, about 18 years of age. Found her suffering with all the agonies of this harassing and obstinate affection. Her mother informed me that she had been suffering about twelve hours, and that this was her third attack this spring. Her pulse was 88; tongue coated, with a white fur; bowels torpid; skin slightly yellow showing very clearly that Miss C.'s face-ache was dependent on a derangement of the hepatic function. Prescribed quinia, sulphas, gr. xii. morphia, acetas, gr. $\frac{1}{2}$, to be taken at once in water, and the affected parts to be rubbed with the following liniment:

℞—Tinct. Opii.

" Belladonna, *aa* ʒiv.

" Camphorae, ℥i.—Misce.

Next morning, quite easy; ordered pil. hydrargyri, gr. iii, to be taken every other night, for two or three weeks. She was free from pain in an hour after taking quinine and morphine, and has continued from that to the present, without any symptom of Tic Doloureux.

Case 4.—*Hemicrania.*—I was called, in the fall of 1852, to see Mrs. ———, a pale, delicate lady. Found her with a pulse 120, small and feeble; tongue slightly coated, and suffering intensely with Hemicrania; she had been bled, blistered and cupped, by her family physician, but without relieving the pain. I learned that she was a dyspeptic, and had been from childhood. On examination, I found her bowels and stomach distended with flatus; skin quite natural. I could not hesitate for a moment, as to the nature of the case; and, in consultation with the attending physician, stated, that the quickness of her pulse was dependent on nervous and not vascular excitement; and her dyspepsia was the remote cause; and the great accumulation of gas in the stomach and bowels, the proximate cause of her nervous headache, and suggested that we give her five grs. of asafœtida, to which he very readily assented. In an hour the evolution of gas became quite audible, which was expelled in great quantities at short intervals, from which time the Hemicrania began to subside. Next morning easy; pulse 100. Prescribed,

℞—Iron by Hydrogen, ℥iiss.

Ext. Cinchonæ.

Pulv. Rhei. *aa* lxxxv.

Hydr. Chlor. Mit. gr. v.

Syrupi, gr. s.

Misce et dividi in pilula, lxxx.

One or two to be taken three times a day, with a wine glass of Porter; the portion to be gradually increased to a pint a day, if she improves, which was done in about a week. In six or eight weeks, I was in the neighborhood, and called to see Mrs. ———, and was pleased to find her in better health than she had been in many years, which she thought; and we believe, entirely due to the stimulating and tonic course of treatment, which is the chief point of interest in the case. An antiphlogistic treatment, as was first adopted by her talented, but young and inexperienced physician, would, I am sure, have agravated the symptoms, and prostrated the patient. (So much for a correct diagnosis.)

CASE 5.—This was a Factory boy, about 12 years of age. When called, we found him complaining of great pain in his feet and ankles, which were greatly swollen. He was unable to walk. As I was in a very great hurry, I did not examine the case, but supposed it to be one of rheumatism, and prescribed the following liniment :

℞—Tinct. opii.

" Belladonnæ.

Pulv. Camphoræ.

Aquæ Ammoniæ.

Olei. Terebinthinæ.

" Sassafras.

" Origani, *aa* ℥ss.

Alcohol, ℥iv.

Misce et signa—Rub the affected parts twice a day, with a woolen cloth, or the palm of the hand.

Requested his father to let me hear from him in two days. At the appointed time, his father reported my patient no better, and desired me to see him again. In a few hours, I called and found the boy in the same position, and his feet and ankles, as his father reported, no better. On examination, I found his abdomen swollen, bowels torpid; skin pale, leucophlegmatic; pulse 100 ; tongue coated and studded, with small pink-colored spots, resembling the top of a pepper castor." I at once pronounced it a case of worms; I discontinued liniment, and prescribed calomel, gr. x, and the following infusion :

℞—Spigelia, ℥i.

Senna, Fol., ʒi

Aq. Bull., Oi.

Fiat infusio signa—A wine glass full to be given every six hours. In 12 hours, he passed 20 or 30 large lumbrici. I saw him no more ; but in a few days he was reported able to walk without pain. Prescribed feri phosph., gr. 5, three times per diem for three weeks; pil. hydrargyri, gr. 3 ounce a week for the same length of time. My patient is now a hearty, ruddy boy.

The chief point of interest in this case is, that worms in the intestines can produce rheumatic symptoms in the lower extremities, as they do other secondary and sympathic symptoms—such as dry cough, headache, cold surface, &c. It may also serve to caution the young practioner against a hasty diagnosis, founded upon a casual examination, and impress his mind with the importance of a careful examination in all cases, however simple and insignificant things may appear at first sight. If we wish to treat a disease scientifically and successfully, we must know its true pathology.

